# Molecular Epidemiology and Antifungal Susceptibility Profile in *Nakaseomyces glabrata* Species Complex: A 5‐Year Countrywide Study

**DOI:** 10.1002/jcla.25042

**Published:** 2024-05-22

**Authors:** Maryam Salimi, Javad Javidnia, Leila Faeli, Azam Moslemi, Mohammad Taghi Hedayati, Iman Haghani, Seyed Reza Aghili, Maryam Moazeni, Parisa Badiee, Maryam Roudbari, Hossein Zarrinfar, Rasoul Mohammadi, Ensieh Lotfali, Sadegh Nouripour‐Sisakht, Seyedmojtaba Seyedmousavi, Tahereh Shokohi, Mahdi Abastabar

**Affiliations:** ^1^ Student Research Committee, School of Medicine Mazandaran University of Medical Sciences Sari Iran; ^2^ Invasive Fungi Research Center, Communicable Diseases Institute Mazandaran University of Medical Sciences Sari Iran; ^3^ Department of Medical Mycology, School of Medicine Mazandaran University of Medical Sciences Sari Iran; ^4^ Clinical Microbiology Research Center Shiraz University of Medical Sciences Shiraz Iran; ^5^ Department of Parasitology and Mycology, School of Medicine Iran University of Medical Sciences Tehran Iran; ^6^ Allergy Research Center Mashhad University of Medical Sciences Mashhad Iran; ^7^ Department of Medical Parasitology and Mycology, School of Medicine Isfahan University of Medical Sciences Isfahan Iran; ^8^ Department of Medical Parasitology and Mycology, School of Medicine Shahid Beheshti University of Medical Sciences Tehran Iran; ^9^ Medicinal Plants Research Center Yasuj University of Medical Sciences Yasuj Iran; ^10^ Microbiology Service, Department of Laboratory Medicine, Clinical Center National Institutes of Health Bethesda Maryland USA

**Keywords:** genotyping, haplotype diversity, multiplex PCR, *Nakaseomyces glabrata*

## Abstract

**Background:**

The current study aimed to identify Iranian *Nakaseomyces* (*Candida*) *glabrata* complex species in the clinical isolates and determine their antifungal susceptibility profile.

**Methods:**

In total, 320 *N. glabrata* clinical isolates were collected from patients hospitalized in different geographical regions of Iran. The initial screening was performed by morphological characteristics on CHROMagar *Candida*. Each isolate was identified by targeting the D1/D2 rDNA using a multiplex‐PCR method. To validate the mPCR method and determine genetic diversity, the ITS‐rDNA region was randomly sequenced in 40 isolates. Additionally, antifungal susceptibility was evaluated against nine antifungal agents following the CLSI M27‐A4 guidelines.

**Results:**

All clinical isolates from Iran were identified as *N. glabrata*. The analysis of ITS‐rDNA sequence data revealed the presence of eight distinct ITS clades and 10 haplotypes among the 40 isolates of *N. glabrata*. The predominant clades identified were Clades VII, V, and IV, which respectively accounted for 22.5%, 17.5%, and 17.5% isolates. The widest MIC ranges were observed for voriconazole (0.016–8 μg/mL) and isavuconazole (0.016–2 μg/mL), whereas the narrowest ranges were seen with itraconazole and amphotericin B (0.25–2 μg/mL).

**Conclusion:**

Haplotype diversity can be a valuable approach for studying the genetic diversity, transmission patterns, and epidemiology of the *N. glabrata* complex.

## Graphical abstract

Because of the increasing prevalence of azole resistance, the treatment of invasive *Nakaseomyces glabrata* infection is considered an important clinical challenge. To determine the source of fungal infection, drug susceptibility, geographical distribution, and any relationships between them, it is necessary and important to determine the genotype. The initial screening was performed by morphological characteristics on CHROMagar *Candida*. Each isolate was identified by targeting the D1/D2 rDNA using a multiplex‐PCR method. To validate the mPCR method and determine genetic diversity, the ITS‐rDNA region was randomly sequenced in 40 isolates. Maximum likelihood phylogeny tree based on DNA sequence data was constructed using DNA sequence data for the ITS‐rDNA region from 40 isolates of *N. glabrata* sensu stricto. The present study revealed the presence of eight distinct ITS clades among the isolates. The predominant clades identified were Clades VII, V, and IV. The widest MIC ranges were observed for voriconazole and isavuconazole, whereas the narrowest ranges were seen with itraconazole and amphotericin B.

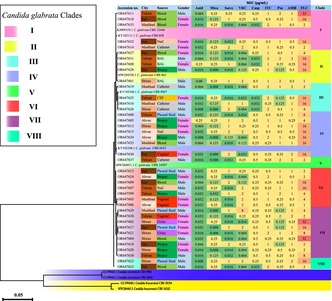

## 1 Introduction

Over 150 yeast species have the potential to cause disease in humans [1]. Nearly 90% of all *Candida* infections are caused by *C. albicans*, *Nakaseomyces* (*Candida*) *glabrata*, *C. parapsilosis*, and *C. tropicalis* [2]. In recent years, non‐*albicans Candida* species, including *N. glabrata*, have been considered important opportunistic pathogens and second rank in invasive candidiasis, which are emerging threats to resistance to fluconazole [3, 4, 5, 6]. *N. glabrata* is known as one of the leading causes of invasive fungal infections because of its high mortality rate [7, 8]. In addition, *C. auris* is an emerging fungus that presents a serious global health threat [9].

A decade ago, advancements in genomic research revealed that *N. glabrata* belongs to the *Nakaseomyces* clade of *Saccharomycotina*, which also includes five other species such as *C. nivariensis* and *C. bracarensis* [10, 11, 12]. Because of several different traits between these cryptic species in their antifungal profile and mortality rate, differentiating them is very important [11]. Recently, a multiplex PCR (mPCR) protocol has been used to distinguish *N. glabrata* from *C. bracarensis* and *C. nivariensis*, which are closely related species [13].

Although echinocandins are now considered a first‐line empirical treatment for invasive *Candida* infections, there has been an increase in resistance to echinocandins in *Candida* species [14, 15, 16, 17]. In addition, considering that more than a decade has passed since the description of these cryptic species, there is still limited information about antifungal susceptibility patterns and distribution in different geographical areas. From an evolutionary standpoint, detecting and identifying these cryptic species can be useful to clarify their pathogenicity [18, 19]. Because of the increasing prevalence of azole resistance, the treatment of invasive *N. glabrata* infection is considered an important clinical challenge [20]. To determine the source of fungal infection, drug susceptibility, geographical distribution, and any relationships between them, it is necessary and important to determine the genotype [21]. Therefore, the present multicenter study aimed to identify clinical isolates of Iranian *N. glabrata* complex species, perform genetic diversity analysis, and characterize their antifungal susceptibility profiles.

## 2 Materials and Methods

### 2.1 Isolates and Phenotypic Identification

In a prospective countrywide study, 320 clinical isolates with phenotypic similarities to *N. glabrata* onto CHROMagar *Candida* (CHROMagar, Paris, France) were collected from patients referred to hospitals and medical centers in Tehran (77 samples), Shiraz (52 samples), Isfahan (25 samples), Mashhad (63 samples), Ahvaz (40 samples), Yasuj (16 samples), and Mazandaran (47 samples) provinces between 2018 and 2023. These isolates were initially recultured on CHROMagar *Candida* medium and identified as a *N. glabrata* species complex.

### 2.2 DNA Extraction

Genomic DNA from *N. glabrata* isolates was extracted using the phenol–chloroform DNA method [22]. For this purpose, the colonies were lysed in 300 μL of lysis buffer (containing 200 mmol/L Tris–HCl [pH 7.5], 25 mmol/L EDTA, 0.5% [w/v] SDS, and 250 mmol/L NaCl), followed by incubation at 100°C for 15 min and then centrifugation. The supernatant was mixed with 20 μL of 3 M sodium acetate, frozen at −20°C for an hour, and centrifuged at 12,000 *g* for 10 min. The supernatants were precipitated with an equal volume of ice‐cold isopropanol, centrifuged at 10,000 *g* for 10 min, washed with ice‐cold 70% ethanol, air‐dried, and resuspended in 50 μL TE buffer. A nanodrop spectrophotometer analysis was performed to assess the quality and quantity of the isolated DNA. Finally, the DNA samples were stored at −20°C until needed.

### 2.3 Multiplex Polymerase Chain Reaction

Multiplex PCR was utilized to identify *N. glabrata* species by targeting the D1/D2 domain of the large subunit ribosomal DNA with specific primers, enabling discrimination between *N. glabrata*, *C. bracarensis*, and *C. nivariensis*, as previously described [23]. The PCR reaction was carried out in a final volume of 25 μL as follows: 8.5 μL of master mix, 12.5 μL of ultrapure water, 1 μL each of *N. glabrata* complex primers, and 1 μL of DNA template. The PCR program included a predenaturation step at 94°C for 5 min, followed by 35 cycles of denaturation at 94°C for 30 s, annealing at 60°C for 30 s, extension at 72°C for 30 s, and a final extension at 72°C for 8 min. PCR products were separated on 2% agarose gels stained with SafeStain (Thermo Fisher Scientific, MA, USA) and subsequently visualized using gel documentation to differentiate between species complexes on the basis of the size of the amplicon. The mPCR is designed to produce amplicons of approximately 386, 214, and 588 bp from *N. glabrata* sensu stricto, *C. bracarensis*, and *C. nivariensis*, respectively.

### 2.4 PCR Sequencing of ITS Region of rDNA

DNA sequencing was performed for the ITS‐rDNA region of 40 randomly selected isolates to confirm the *N. glabrata* species complex and investigate the genetic diversity among the isolated species. For this purpose, the ITS region was amplified using the pan‐fungal ITS1 and ITS4 primers, as described previously [24]. PCR products were sequenced using the Sanger method in the forward direction. Nucleotide BLAST analysis was performed on the resulting sequences from each isolate by comparing them to sequences in the NCBI database. Accurate species identification requires a minimum sequence identity of at least 99% for reference sequences. The obtained sequences were submitted to the GenBank database.

### 2.5 Phylogenetic Analysis

The clade and variation among the *N. glabrata* isolates were studied by comparing ITS‐rDNA region sequences. The full length of sequences was cut and trimmed as required using MEGA 11 software. Multiple sequence alignments were carried out to compare the 40 randomly selected *N. glabrata* isolate sequences.

Finally, a phylogenetic tree was constructed with the maximum likelihood phylogeny method in MEGA 11 software and 1000 bootstraps [25]. To draw a phylogenetic tree of six type strains of *N. glabrata* (CBS 12440, CBS 858, CBS 862, CBS 8947, CBS 6633, CBS 14397), two type strains of *C. nivariensis* (CBS 9983, CBS 10161), and two type strains of *C. bracarensis* (CBS 10154, CBS 16242).

### 2.6 ITS‐rDNA Haplotype Diversity Analysis

To assess the predominant ITS haplotypes, determine intraspecies differences, and assess genetic diversity, an analysis of haplotype diversity was conducted. Pairwise comparisons and multiple sequence alignments were performed using CLUSTAL W2. For this purpose, a set of 40 randomly selected *N. glabrata* species were used, and their nucleotide sequences of ITS‐rDNA were aligned using the MEGA 11 software. The aligned sequences were exported as a Nexus format file. Then, the NEXUS format was used in the PopART v. 1.7 software to prepare visualizations of the haplotype networking and genetic distance among Iranian *N. glabrata* species complex isolates [26].

### 2.7 Antifungal Susceptibility Testing

Broth microdilution was used to perform in vitro antifungal susceptibility testing in accordance with the protocols outlined in Clinical and Laboratory Standards Institute (CLSI) M27‐A4 and M60 guidelines [27, 28]. The in vitro activity of nine antifungal drugs, including itraconazole (Janssen Pharmaceutical, Beers, Belgium), anidulafungin, fluconazole, isavuconazole, posaconazole, voriconazole (Pfizer), amphotericin B (Bristol‐Myers‐Squib, Quebec, Canada), caspofungin (Merck KGaA, Darmstadt, Germany), and micafungin (Astellas Pharma Inc., Tokyo, Japan), against *N. glabrata* isolates was assessed.

The wells contained antifungal drugs at final concentrations ranging from 0.016 to 16 μg/mL for voriconazole, itraconazole, amphotericin B, isavuconazole, and posaconazole, 0.064–64 μg/mL for fluconazole, and 0.008–8 μg/mL for anidulafungin, caspofungin, and micafungin.

To ensure quality control and reproducibility of the testing methods, American Type Culture Collection (ATCC) control strains (*C. krusei* ATCC 6258 and *C. parapsilosis* ATCC 22019) were included. The minimum inhibitory concentrations (MICs) were interpreted on the basis of the proposed CLSI M27‐A4 and CLSI M60 breakpoints [27, 28].

### 2.8 Statistical Analysis

The data, including the ITS clade, source of *N. glabrata* species complex isolation, geographic location of the isolates' origin, and MIC of the isolates, were transferred to IBM SPSS Statistics software version 24. The probability of isolating a distinct ITS type with respect to the type of *N. glabrata*, location of isolation, and MIC were computed using the chi‐squared test. An alpha level of 0.05 was used as a statistical significance criterion for all statistical tests.

## 3 Results

### 3.1 Phenotypic Identification of *Nakaseomyces glabrata* Complex Isolates

The purity of 320 clinical isolates was verified by their growth on CHROMagar *Candida* medium. Table 1 shows different clinical samples among the 320 identified *N. glabrata* complexes. The blood sample had the highest sample count at 23.1%, whereas CSF had the lowest count at 0.93%.

**TABLE 1:** Different clinical samples among 320 identified *Nakaseomyces glabrata* isolates.

Sample type	Number (%)
Blood	74 (23.1)
Biopsy	57 (17.81)
Vaginal	43 (13.43)
Urine/renal sample	41 (12.81)
Catheter	38 (11.87)
Nail	29 (9.06)
Bronchoalveolar lavage fluid (BAL)	18 (5.62)
Pleural fluid	17 (5.31)
Cerebrospinal fluid (CSF)	3 (0.93)
Total	320 (100)

### 3.2 Establishment of mPCR Assay and Analysis of Clinical *Nakaseomyces glabrata* Isolates

All 320 isolates produced ~386 bp amplicons, which is characteristic of *N. glabrata* sensu stricto strains. None of the clinical isolates produced the 588 and 214 bp amplicons that are the characteristics of *C. nivariensis* and *C. bracarensis*, respectively. The accession numbers for the 40 ITS sequences were released in the NCBI GenBank nucleotide database (https://www.ncbi.nlm.nih.gov/nuccore) under accession numbers OR647599–OR647638.

### 3.3 Phylogenetic Analysis

**FIGURE 1:**
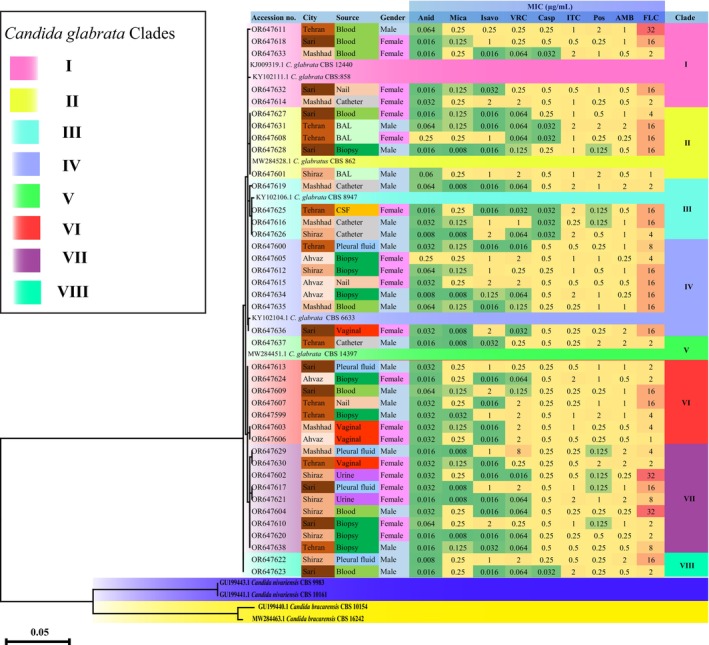
Maximum likelihood phylogeny tree based on DNA sequence data for the ITS region of rDNA from 40 *Nakaseomyces glabrata* sensu stricto isolates. The associated taxa clustered together in the bootstrap test (1000 replicates) are shown next to the branches.

On the basis of ITS region phylogenetic analysis data, eight distinct clades (arbitrarily assigned as I–VIII) were identified among 40 clinical *N. glabrata* isolates. Figure 1 illustrates a maximum likelihood phylogenetic tree constructed using DNA sequence data for the ITS‐rDNA region from a total of 40 isolates of *N. glabrata* sensu stricto. On the basis of the findings from the phylogenetic tree (Figure 1), a total of eight distinct clades were identified. Among these clades, Clade VII accounted for 22.5% of the isolates, followed by Clade VI and Clade IV, each representing 17.5% of the isolates. Clades I and II accounted for 12.5% each, whereas Clade III represented 10% of the isolates. Clade VIII was observed in 5% of the isolates, and Clade V was found in 2.5% of the isolates.

**FIGURE 2:**
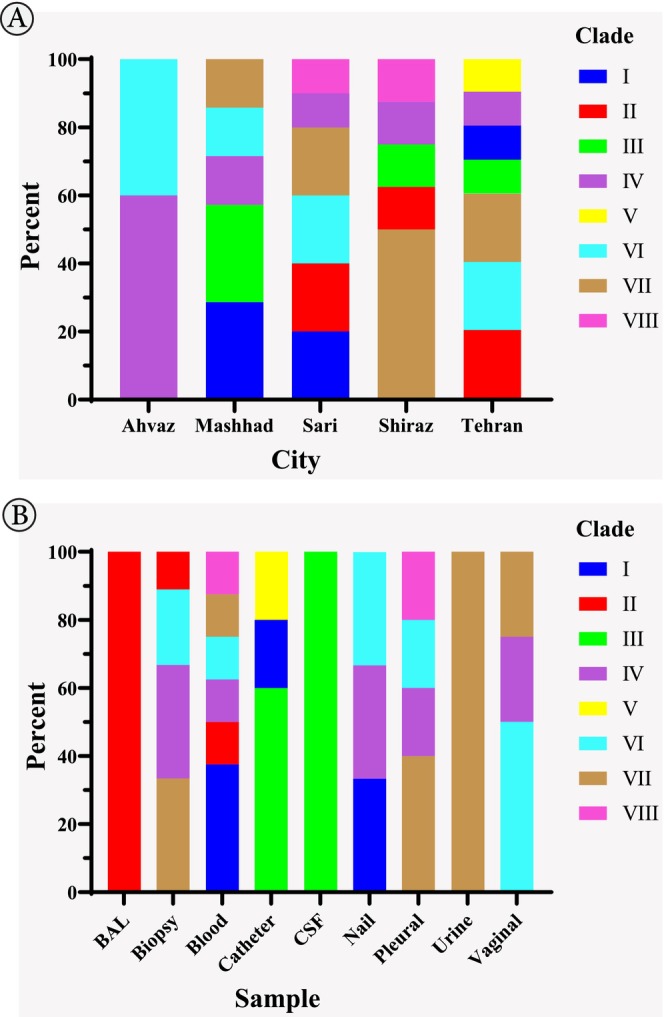
Genetic diversity of 40 *Nakaseomyces glabrata* sensu stricto isolates based on city (A) and sample sources (B).

Figure 2 demonstrates the genetic diversity analysis of 40 *N. glabrata* sensu stricto isolates, focusing on two aspects: city (A) and sample sources (B). Tehran had the highest number of clades, with a total of seven clades identified among the isolates (Figure 2A). On the contrary, the city of Ahvaz had the lowest number of clades, with only two clades observed. Clade V was exclusively isolated in Tehran and was also identified in isolates obtained from catheters. Clade V was exclusively detected in samples collected from Tehran, indicating its specific presence within this geographical location. On the contrary, Clade IV was identified in isolates from all cities under investigation, suggesting a wider distribution across different geographical regions. These findings highlight the geographical specificity of Clade V to Tehran and the broader presence of Clade IV across all cities.

Clade II was exclusively identified in 100% of BAL samples, whereas Clade VII was solely detected in 100% of urine samples (Figure 2B). Additionally, Clade III was exclusively present in 100% of CSF samples. A statistically significant relationship was detected between clade and sample type, as indicated by a *p* value of 0.01. Among women, Clade VII was the most prevalent, whereas Clade VI was the most common among men. No statistically significant relationship was found between clade and gender (*p* value = 0.4).

### 3.4 ITS Haplotypes Among *Nakaseomyces glabrata* Isolates

**FIGURE 3:**
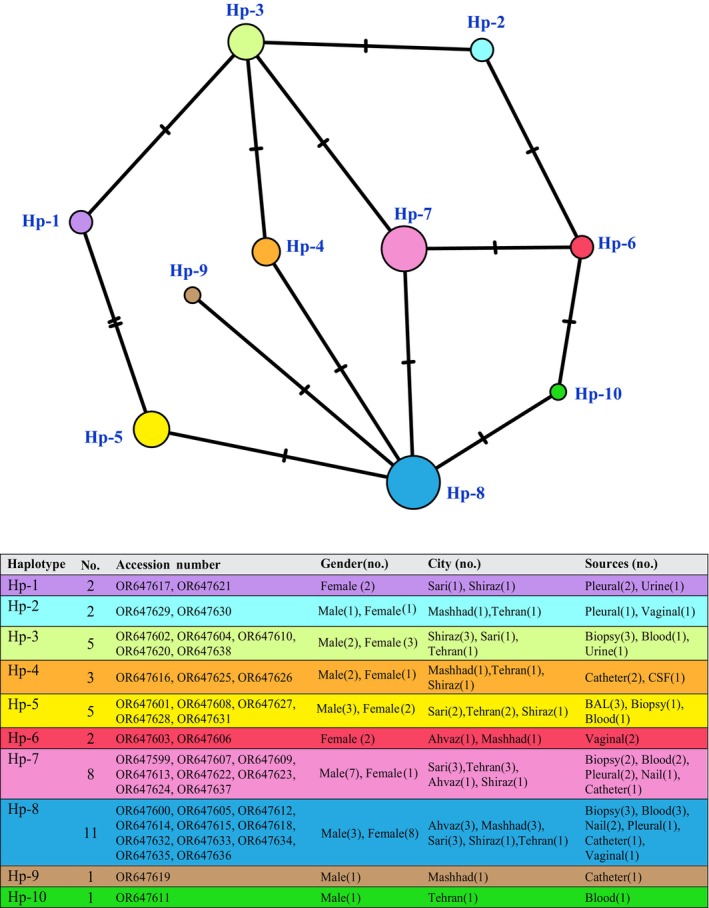
Haplotype network generated for Internal transcribed spacer (ITS) region sequences on 40 *Nakaseomyces glabrata* sensu stricto isolates. The size of the circle indicates the relative frequency of sequences belonging to a particular haplotype (smallest circle = 1 sequence to largest circle = 11 sequences). Hatch markers along the network branches indicate the number of SNP.

Regarding the ITS sequence, 10 haplotypes were detected on the basis of the sequence analysis of 40 isolates obtained from different cities. Investigation of haplotype diversity within the strains led to the identification of 10 haplotypes, six of which were dominant and two of which were common. The largest haplotype groups were related to Haplotypes 8 and 7, which are related to Clades I, IV, V, VI, and VIII, which belonged to the populations of Tehran, Mashhad, Ahvaz, Shiraz, and Sari (Figure 3). A network analysis was performed on the haplotypes found during the sequencing process to validate the results obtained from the genetic distance matrix method.

The haplotyping analysis demonstrated a nucleotide diversity of 0.0025 between different strains of *N. glabrata*. Generally, the results indicated a high rate of genetic diversity and haplotype diversity. Analysis of ITS sequences revealed that both indices (*π* = 0.0025) were high. Tests of the standard neutral model for a demographically stable population‐Tajima's *D* (0.730) were carried out on the ITS sequences. The neutrality statistics were negative and not significant at *p* = 0.47, perhaps due to the intronic regions present in the ITS sequences.

### 3.5 Antifungal Susceptibility Testing

All isolates included in the study exhibited sensitivity to echinocandins. Three isolates belonging to Clades I and VII exhibited high Minimum Inhibitory Concentration (MIC) values for fluconazole. Among all the clades, fluconazole was the only drug that demonstrated relatively high MIC values.

Table 2 presents a concise summary of the findings from the antifungal susceptibility testing, including the geometric mean (GM) minimum inhibitory concentrations (MICs) and minimum effective concentrations (MECs), the MIC/MEC ranges, as well as the MIC50/MEC50 and MIC90/MEC90 distributions of the tested drugs. The widest MIC/MEC ranges were observed for voriconazole (0.016–8 μg/mL) and isavuconazole (0.016–2 μg/mL), whereas the narrowest ranges were observed for itraconazole and amphotericin B (0.25–2 μg/mL). Anidulafungin (0.02 μg/mL) and micafungin (0.08 μg/mL) had the lowest GM MIC/MEC values, followed by isavuconazole (0.12 μg/mL), voriconazole (0.23 μg/mL), caspofungin (0.24 μg/mL), posaconazole (0.47 μg/mL), itraconazole (0.72 μg/mL), amphotericin B (0.76 μg/mL), and fluconazole (6.69 μg/mL).

**TABLE 2:** In vitro susceptibilities of nine antifungal drugs against 40 clinical strains of *Nakaseomyces glabrata*.

Antifungal drug	MIC parameter (μg/mL)				
	MIC_50_	MIC_90_	MIC range	G mean	Mode
Amphotericin B	1	2	0.25–2	0.758	1
Fluconazole	8	16	1–32	6.727	16
Itraconazole	1	2	0.25–2	0.719	1
Voriconazole	0.125	2	0.016–8	0.228	0.064
Posaconazole	0.5	2	0.125–2	0.467	0.25
Isavuconazole	0.032	2	0.016–2	0.122	0.016
Caspofungin	0.25	0.5	0.032–0.5	0.247	0.5
Anidulafungin	0.032	0.064	0.008–0.25	0.029	0.032
Micafungin	0.125	0.25	0.008–0.25	0.082	0.25

Abbreviations: G mean, geometric mean; MIC, minimum inhibitory concentrations.

## 4 Discussion

*Nakaseomyces glabrata* is widely recognized as one of the primary causes of invasive candidiasis, a condition that is becoming an increasingly significant public health concern globally. Two cryptic species, *C. bracarensis* and *C. nivariensis*, are not distinguishable from *N. glabrata* [11, 29]. Molecular identification and differentiation between these species' complexes are crucial because of variations in antifungal susceptibility and virulence profiles [1, 18, 30]. Because of the differences in mortality rate and antifungal susceptibility among *N. glabrata* complex species, as well as the lack of comprehensive epidemiological data and conflicting findings in various studies, the antifungal susceptibility profile and geographic distribution of these cryptic species are important. Therefore, accurate identification of the species in this complex is necessary for proper antifungal treatment [1, 31, 32]. In relation to this matter, techniques such as multiplex PCR enable quick, affordable, precise, and reliable identification of cryptic species of *N. glabrata*. In spite of *C. nivariensis* and *C. bracarensis* reports from different countries, limited information has been provided about their antifungal susceptibility patterns, epidemiology, actual prevalence, and biological niches. Therefore, the present study was conducted with the aim of identifying and determining the clinical characteristics, antifungal susceptibility, and clade and haplotype of the *N. glabrata* species complex in Iran. In the present study, 320 isolates collected from various regions of Iran were analyzed, and none of them were identified as *C. bracarensis* or *C. nivariensis*. These findings align with recent research, in which similarly, Esposto et al. [33] investigated 1000 clinical isolates obtained from 14 regions in Italy but did not detect any instances of *C. nivariensis* or *C. bracarensis*. Furthermore, in a study conducted in Kuwait, among 440 clinical isolates, 100% of the isolates were reported as *N. glabrata* sensu stricto [34]. In a study of 143 clinical isolates collected in Spain, three samples (2%) were identified as *C. bracarensis*, whereas no isolates were identified as *C. nivariensis* [35]. Additionally, Angoulvant, Guitard and Hennequin. [8] reported the prevalence of these two cryptic species as *NC. bracarensis* (0.01%) and *C. nivariensis* (0.12%). Various molecular techniques have been previously documented for the identification of isolates specific to *N. glabrata* sensu stricto, *C. nivariensis*, and/or *C. bracarensis* [13, 30, 36, 37]. Among these methods, similar to our study, Arastehfar et al. [23] and Asadzadeh et al. [34] utilized a simple, fast, and cost‐effective method on the basis of mPCR for the accurate identification of the *N. glabrata* complex, which we confirm as well. Moreover, according to the present study, no isolates of *C. nivariensis* and *C. bracarensis* were isolated, indicating the low prevalence of these cryptic species in Iran.

Given that there is considerable variation in virulence patterns among *N. glabrata* strains, as well as associations between specific clades and increased mortality rates, the critical role of genotyping techniques in diagnosis has been highlighted [38, 39].

Considering that few studies have been done on the genotyping of the *N. glabrata* complex and there is little information about it, two studies close to ours can be mentioned in this regard. Asadzadeh et al. [34] in Kuwait, 85 isolates of *N. glabrata* were randomly selected from among 440 isolates using ITS and sequencing. Finally, 28 haplotypes were identified, of which 18 isolates had distinct clades and 67 isolates were grouped into 10 haplotypes. In this study, 40 samples were randomly selected from a total of 320 *N. glabrata* isolates, resulting in the identification of eight clades and nine haplotypes. The variation in the number of clades and haplotypes observed in different studies may be attributed to factors such as the number of samples, duration of sample collection, and inclusion of samples from diverse sources. These findings support the high degree of heterogeneity among the *N. glabrata* species complex in Iran (Tehran, Shiraz, Yasuj, Isfahan, Ahvaz, Mashhad, and Mazandaran provinces), despite the same source of samples and geographical locations, suggesting that isolates from patients originated from another side during hospitalization. The strength of our study is that it is the first investigation of the clade of the Iranian species complex of *N. glabrata* from Tehran, Shiraz, Ahvaz, Mashhad, and Mazandaran provinces. In order to assess the genetic diversity and transmission patterns of the *N. glabrata* species complex, genotyping is essential for epidemiological investigations, in particular where patients are being treated with different treatments. The limitation of this study is limited to the number of isolates analyzed and a lack of information about ITS sequences for *N. glabrata* species collected in Iran.

There is not sufficient data to support the hypothesis that all species of the *N. glabrata* complex are generally susceptible to echinocandins. In light of different susceptibility patterns among the *N. glabrata* species complex, the lower activity of echinocandin was reported against *C. nivariensis* [40]. On the basis of the findings of different studies and various drug susceptibility patterns of the *N. glabrata* complex, accurate identification of these species is very important [41]. According to a study conducted by Hou et al. [18] using molecular methods, 12 isolates of *C. nivariensis* and 1 isolate of *C. bracarensis* were identified, and 100% of them were susceptible to echinocandins by antifungal susceptibility evaluation. According to the findings of this study, *N. glabrata* sensu stricto isolates showed resistance to caspofungin and micafungin while being sensitive to anidulafungin. These findings are consistent with previous studies. It is noteworthy that recent evidence casts doubt on the future effectiveness of echinocandins, which are a class of antifungal drugs. Several studies have reported resistance to azoles in these two species [1, 30, 40, 41, 42]. The most striking result in our study was that all *N. glabrata* had a low MIC value for itraconazole, voriconazole, and posaconazole; all the isolates were found to be susceptible to echinocandins, whereas 7.5% of the samples demonstrated resistance to fluconazole. According to various reports, as there is a significant difference between these two species in terms of susceptibility to antifungal drugs compared with *N. glabrata* senso stricto, studies with larger sample sizes in different geographic locations should be conducted to clarify their epidemiology and antifungal susceptibility profile [30]. Our ability to identify and share data on a national and global scale will help us learn about these species and improve diagnosis and treatment. It can also aid in understanding the evolution and spread of this fungal pathogen.

## 5 Conclusions

Detection and identification of cryptic *N. glabrata* species in relation to their closely related counterparts present challenges that have led to uncertainties concerning their susceptibility to antifungal agents and their epidemiology. In addition, because of the importance of identifying these species with the shortest turnaround time, it is very necessary to develop cost‐effective, efficient, and reliable methods. Given the documented increase of these cryptic species in multiple European countries and their propensity to develop resistance to antifungal treatments, it is recommended to continue monitoring these emerging cryptic species.

## Conflicts of Interest

The authors declare no conflicts of interest.

## Data Availability

The data that support the findings of this study are openly available in the NCBI GenBank nucleotide database at https://www.ncbi.nlm.nih.gov/nuccore, under accession numbers OR647599–OR647638.

## References

[1] A. M. Borman , R. Petch , C. J. Linton , M. D. Palmer , P. D. Bridge , and E. M. Johnson , “ *Candida nivariensis*, an Emerging Pathogenic Fungus With Multidrug Resistance to Antifungal Agents,” Journal of Clinical Microbiology 46, no. 3 (2008): 933–938. 18199788 10.1128/JCM.02116-07 PMC2268332

[2] M. A. Pfaller , D. J. Diekema , D. L. Gibbs , et al., “Results From the ARTEMIS DISK Global Antifungal Surveillance Study, 1997–2007: 10.5‐Year Analysis of Susceptibilities of Noncandidal Yeast Species to Fluconazole and Voriconazole Determined by CLSI Standardized Disk Diffusion Testing,” Journal of Clinical Microbiology 47, no. 1 (2009): 117–123. 19005141 10.1128/JCM.01747-08 PMC2620874

[3] M. E. Falagas , N. Roussos , and K. Z. Vardakas , “Relative Frequency of Albicans and the Various Non‐albicans *Candida* spp. Among Candidemia Isolates From Inpatients in Various Parts of the World: A Systematic Review,” International Journal of Infectious Diseases 14, no. 11 (2010): e954–e966. 20797887 10.1016/j.ijid.2010.04.006

[4] P. L. Fidel, Jr., J. A. Vazquez , and J. D. Sobel , “ *Candida glabrata*: Review of Epidemiology, Pathogenesis, and Clinical Disease With Comparison to *C. albicans* ,” Clinical Microbiology Reviews 12, no. 1 (1999): 80–96. 9880475 10.1128/cmr.12.1.80 PMC88907

[5] M. A. Pfaller and D. J. Diekema , “Epidemiology of Invasive Candidiasis: A Persistent Public Health Problem,” Clinical Microbiology Reviews 20, no. 1 (2007): 133–163. 17223626 10.1128/CMR.00029-06 PMC1797637

[6] W. Trick , S. Fridkin , J. Edwards , R. Hajjeh , R. P. Gaynes , and National Nosocomial Infections Surveillance System Hospitals , “Secular Trend of Hospital‐Acquired Candidemia Among Intensive Care Unit Patients in the United States During 1989–1999,” Clinical Infectious Diseases 35, no. 5 (2002): 627–630. 12173140 10.1086/342300

[7] D. Diekema , S. Arbefeville , L. Boyken , J. Kroeger , and M. Pfaller , “The Changing Epidemiology of Healthcare‐Associated Candidemia Over Three Decades,” Diagnostic Microbiology and Infectious Disease 73, no. 1 (2012): 45–48. 22578938 10.1016/j.diagmicrobio.2012.02.001

[8] A. Angoulvant , J. Guitard , and C. Hennequin , “Old and New Pathogenic Nakaseomyces Species: Epidemiology, Biology, Identification, Pathogenicity and Antifungal Resistance,” FEMS Yeast Research 16, no. 2 (2016): fov114. 26691882 10.1093/femsyr/fov114

[9] A. Chowdhary , C. Sharma , and J. F. Meis , “ *Candida auris*: A Rapidly Emerging Cause of Hospital‐Acquired Multidrug‐Resistant Fungal Infections Globally,” PLoS Pathogens 13, no. 5 (2017): e1006290. 28542486 10.1371/journal.ppat.1006290 PMC5436850

[10] T. Saraya , K. Tanabe , K. Araki , et al., “Breakthrough Invasive *Candida glabrata* in Patients on Micafungin: A Novel FKS Gene Conversion Correlated With Sequential Elevation of MIC,” Journal of Clinical Microbiology 52, no. 7 (2014): 2709–2712. 24789192 10.1128/JCM.03593-13 PMC4097732

[11] J. Alcoba‐Florez , S. Mendez‐Alvarez , J. Cano , J. Guarro , E. Perez‐Roth , and M. del Pilar Arevalo , “Phenotypic and Molecular Characterization of *Candida nivariensis* sp. nov., a Possible New Opportunistic Fungus,” Journal of Clinical Microbiology 43, no. 8 (2005): 4107–4111. 16081957 10.1128/JCM.43.8.4107-4111.2005 PMC1233986

[12] S. R. Lockhart , S. A. Messer , M. Gherna , et al., “Identification of *Candida nivariensis* and *Candida bracarensis* in a Large Global Collection of *Candida glabrata* Isolates: Comparison to the Literature,” Journal of Clinical Microbiology 47, no. 4 (2009): 1216–1217. 19193845 10.1128/JCM.02315-08 PMC2668319

[13] O. Romeo , F. Scordino , I. Pernice , C. Lo Passo , and G. Criseo , “A Multiplex PCR Protocol for Rapid Identification of *Candida glabrata* and Its Phylogenetically Related Species *Candida nivariensis* and *Candida bracarensis* ,” Journal of Microbiological Methods 79, no. 1 (2009): 117–120. 19635503 10.1016/j.mimet.2009.07.016

[14] S. R. Lockhart , N. Iqbal , A. A. Cleveland , et al., “Species Identification and Antifungal Susceptibility Testing of Candida Bloodstream Isolates From Population‐Based Surveillance Studies in Two US Cities From 2008 to 2011,” Journal of Clinical Microbiology 50, no. 11 (2012): 3435–3442. 22875889 10.1128/JCM.01283-12 PMC3486211

[15] S. Katiyar , M. Pfaller , and T. Edlind , “ *Candida albicans* and *Candida glabrata* Clinical Isolates Exhibiting Reduced Echinocandin Susceptibility,” Antimicrobial Agents and Chemotherapy 50, no. 8 (2006): 2892–2894. 16870797 10.1128/AAC.00349-06 PMC1538661

[16] E. Dannaoui , M. Desnos‐Ollivier , D. Garcia‐Hermoso , et al., “ *Candida* spp. With Acquired Echinocandin Resistance, France, 2004–2010,” Emerging Infectious Diseases 18, no. 1 (2012): 86–90. 22257484 10.3201/eid1801.110556 PMC3310099

[17] B. D. Alexander , M. D. Johnson , C. D. Pfeiffer , et al., “Increasing Echinocandin Resistance in *Candida glabrata*: Clinical Failure Correlates With Presence of FKS Mutations and Elevated Minimum Inhibitory Concentrations,” Clinical Infectious Diseases 56, no. 12 (2013): 1724–1732. 23487382 10.1093/cid/cit136 PMC3658363

[18] X. Hou , M. Xiao , S. C. Chen , et al., “Identification and Antifungal Susceptibility Profiles of *Candida nivariensis* and *Candida bracarensis* in a Multi‐Center Chinese Collection of Yeasts,” Frontiers in Microbiology 8 (2017): 5. 28154553 10.3389/fmicb.2017.00005 PMC5243801

[19] A. Arastehfar , W. Fang , W. Pan , et al., “YEAST PANEL Multiplex PCR for Identification of Clinically Important Yeast Species: Stepwise Diagnostic Strategy, Useful for Developing Countries,” Diagnostic Microbiology and Infectious Disease 93, no. 2 (2019): 112–119. 30377018 10.1016/j.diagmicrobio.2018.09.007

[20] P. G. Pappas , C. A. Kauffman , D. Andes , et al., “Clinical Practice Guidelines for the Management of Candidiasis: 2009 Update by the Infectious Diseases Society of America,” Clinical Infectious Diseases 48, no. 5 (2009): 503–535. 19191635 10.1086/596757 PMC7294538

[21] S. Amanloo , M. Shams‐Ghahfarokhi , M. Ghahri , and M. Razzaghi‐Abyaneh , “Genotyping of Clinical Isolates of *Candida glabrata* From Iran by Multilocus Sequence Typing and Determination of Population Structure and Drug Resistance Profile,” Medical Mycology 56, no. 2 (2018): 207–215. 28482076 10.1093/mmy/myx030

[22] M. Kumar and M. Mugunthan , “Evaluation of Three DNA Extraction Methods From Fungal Cultures,” Medical Journal, Armed Forces India 74, no. 4 (2018): 333–336. 30449918 10.1016/j.mjafi.2017.07.009 PMC6224647

[23] A. Arastehfar , W. Fang , W. Pan , W. Liao , L. Yan , and T. Boekhout , “Identification of Nine Cryptic Species of *Candida albicans*, *C. glabrata*, and *C. parapsilosis* Complexes Using One‐Step Multiplex PCR,” BMC Infectious Diseases 18 (2018): 480. 30253748 10.1186/s12879-018-3381-5 PMC6156947

[24] T. J. White , T. Bruns , S. Lee , and J. Taylor , “Amplification and Direct Sequencing of Fungal Ribosomal RNA Genes for Phylogenetics,” PCR Protocols: A Guide to Methods and Applications 18, no. 1 (1990): 315–322.

[25] M. Asadzadeh , S. Ahmad , N. Al‐Sweih , and Z. Khan , “Population Structure and Molecular Genetic Characterization of 5‐Flucytosine‐Susceptible and ‐Resistant Clinical *Candida dubliniensis* Isolates From Kuwait,” PLoS One 12, no. 4 (2017): e0175269. 28380072 10.1371/journal.pone.0175269 PMC5381908

[26] J. W. Leigh and D. Bryant , “POPART: Full‐Feature Software for Haplotype Network Construction,” Methods in Ecology and Evolution 6, no. 9 (2015): 1110–1116.

[27] CLSI , Reference Method for Broth Dilution Antifungal Susceptibility Testing of Yeasts, M27‐A3 (Wayne, PA: Clinical and Laboratory Standards Institute, 2008).

[28] CLSI , M60. Performance Standards for Antifungal Susceptibility Testing of Yeasts, 1st ed. (Wayne, PA: Clinical and Laboratory Standards Institute, 2017).

[29] A. Correia , P. Sampaio , S. James , and C. Pais , “ *Candida bracarensis* sp. nov., a Novel Anamorphic Yeast Species Phenotypically Similar to *Candida glabrata* ,” International Journal of Systematic and Evolutionary Microbiology 56, no. Pt 1 (2006): 313–317. 16403904 10.1099/ijs.0.64076-0

[30] J. A. Bishop , N. Chase , S. S. Magill , C. P. Kurtzman , M. J. Fiandaca , and W. G. Merz , “ *Candida bracarensis* Detected Among Isolates of *Candida glabrata* by Peptide Nucleic Acid Fluorescence In Situ Hybridization: Susceptibility Data and Documentation of Presumed Infection,” Journal of Clinical Microbiology 46, no. 2 (2008): 443–446. 18077641 10.1128/JCM.01986-07 PMC2238114

[31] A. Gacser , W. Schafer , J. S. Nosanchuk , S. Salomon , and J. D. Nosanchuk , “Virulence of *Candida parapsilosis*, *Candida orthopsilosis*, and *Candida metapsilosis* in Reconstituted Human Tissue Models,” Fungal Genetics and Biology 44, no. 12 (2007): 1336–1341. 17391997 10.1016/j.fgb.2007.02.002

[32] A. M. Borman , A. Szekely , C. J. Linton , M. D. Palmer , P. Brown , and E. M. Johnson , “Epidemiology, Antifungal Susceptibility, and Pathogenicity of Candida Africana Isolates From the United Kingdom,” Journal of Clinical Microbiology 51, no. 3 (2013): 967–972. 23303503 10.1128/JCM.02816-12 PMC3592039

[33] M. Esposto , A. Prigitano , O. Romeo , et al., “Looking for *Candida nivariensis* and *C. bracarensis* Among a Large Italian Collection of *C. glabrata* Isolates: Results of the FIMUA Working Group,” Mycoses 56, no. 3 (2013): 394–396. 23170902 10.1111/myc.12026

[34] M. Asadzadeh , A. F. Alanazi , S. Ahmad , N. Al‐Sweih , and Z. Khan , “Lack of Detection of *Candida nivariensis* and *Candida bracarensis* Among 440 Clinical *Candida glabrata* Sensu Lato Isolates in Kuwait,” PLoS One 14, no. 10 (2019): e0223920. 31618264 10.1371/journal.pone.0223920 PMC6795469

[35] M. Cuenca‐Estrella , A. Gomez‐Lopez , G. Isla , et al., “Prevalence of *Candida bracarensis* and *Candida nivariensis* in a Spanish Collection of Yeasts: Comparison of Results From a Reference Centre and From a Population‐Based Surveillance Study of Candidemia,” Medical Mycology 49, no. 5 (2011): 525–529. 21198347 10.3109/13693786.2010.546373

[36] J. Alcoba‐Florez , P. Arevalo Mdel , F. J. Gonzalez‐Paredes , et al., “PCR Protocol for Specific Identification of *Candida nivariensis*, a Recently Described Pathogenic Yeast,” Journal of Clinical Microbiology 43, no. 12 (2005): 6194–6196. 16333128 10.1128/JCM.43.12.6194-6196.2005 PMC1317211

[37] A. Enache‐Angoulvant , J. Guitard , F. Grenouillet , et al., “Rapid Discrimination Between *Candida glabrata*, *Candida nivariensis*, and *Candida bracarensis* by Use of a Singleplex PCR,” Journal of Clinical Microbiology 49, no. 9 (2011): 3375–3379. 21752976 10.1128/JCM.00688-11 PMC3165624

[38] E. Lopez‐Fuentes , G. Gutierrez‐Escobedo , B. Timmermans , P. Van Dijck , A. De Las Penas , and I. Castano , “ *Candida glabrata*'s Genome Plasticity Confers a Unique Pattern of Expressed Cell Wall Proteins,” Journal of Fungi (Basel) 4, no. 2 (2018): 67. 10.3390/jof4020067 PMC6023349 29874814

[39] S. A. Byun , E. J. Won , M. N. Kim , et al., “Multilocus Sequence Typing (MLST) Genotypes of *Candida glabrata* Bloodstream Isolates in Korea: Association With Antifungal Resistance, Mutations in Mismatch Repair Gene (Msh2), and Clinical Outcomes,” Frontiers in Microbiology 9 (2018): 1523. 30057573 10.3389/fmicb.2018.01523 PMC6053515

[40] M. H. Figueiredo‐Carvalho , S. Ramos Lde , L. S. Barbedo , et al., “First Description of *Candida nivariensis* in Brazil: Antifungal Susceptibility Profile and Potential Virulence Attributes,” Memórias do Instituto Oswaldo Cruz 111, no. 1 (2016): 51–58. 26814644 10.1590/0074-02760150376 PMC4727436

[41] S.‐i. Fujita , Y. Senda , T. Okusi , et al., “Catheter‐Related Fungemia Due to Fluconazole‐Resistant *Candida nivariensis* ,” Journal of Clinical Microbiology 45, no. 10 (2007): 3459–3461. 17652473 10.1128/JCM.00727-07 PMC2045379

[42] R. L. Gorton , G. L. Jones , C. C. Kibbler , and S. Collier , “ *Candida nivariensis* Isolated From a Renal Transplant Patient With Persistent Candiduria‐Molecular Identification Using ITS PCR and MALDI‐TOF,” Medical Mycology Case Reports 2 (2013): 156–158. 24432244 10.1016/j.mmcr.2013.10.001 PMC3885950

